# Symptomatic Outcomes After Autologous Fat Grafting in Irradiated Postmastectomy Chest Wall

**DOI:** 10.3390/healthcare14020281

**Published:** 2026-01-22

**Authors:** Razvan George Bogdan, Mara Nicolau, Alina Helgiu, Zorin Petrisor Crainiceanu

**Affiliations:** 1 Doctoral School, “Victor Babeș” University of Medicine and Pharmacy, 300041 Timișoara, Romania; razvan.bogdan@umft.ro; 2 County Clinical Emergency Hospital “Pius Brînzeu”, 300723 Timișoara, Romania; 3 Faculty of Medicine, “Lucian Blaga” University of Sibiu, 550024 Sibiu, Romania; 4 County Clinical Emergency Hospital of Sibiu, 550245 Sibiu, Romania; 5 Plastic Surgery Department, “Victor Babeș” University of Medicine and Pharmacy, 300041 Timișoara, Romania

**Keywords:** autologous fat grafting, lipofilling, irradiated chest wall, postmastectomy reconstruction, patient reported outcomes, radiation induced fibrosis

## Abstract

**Highlights:**

**What are the main findings?**

- Autologous fat grafting reduced patient-reported symptoms in irradiated postmastectomy chest wall tissue at 6 months.
- The largest descriptive improvements were observed for erythema, burning sensation and pruritus, without postoperative complications.

**What are the implications of the main findings?**

- Symptom based evaluation captures clinically relevant benefits of fat grafting that are not reflected by imaging alone.
- Autologous fat grafting can be considered a safe adjunctive option for symptomatic relief in selected irradiated postmastectomy patients.

**Abstract:**

Background/Objectives: Radiotherapy of the chest wall after mastectomy frequently leads to fibrosis, reduced tissue elasticity, erythema, pain and chronic skin-related symptoms that complicate reconstructive strategies. Autologous fat grafting has been proposed as a regenerative option for radiation induced soft tissue damage, but clinical data focused on patient-reported symptoms remain limited. The objective of this study was to describe symptomatic and clinical changes after autologous fat grafting in irradiated postmastectomy chest wall tissue. Methods: This pilot observational study included five female patients with a history of mastectomy followed by adjuvant chest wall radiotherapy. All patients underwent a single session of standard autologous fat grafting without adipose derived stem cell enrichment. Patient-reported symptoms, including pruritus, local discomfort, burning sensation and erythema, were recorded preoperatively and at six months using a standardized 0 to 5 scale. Scar pliability was assessed by two experienced physicians using the same scale. Only descriptive statistical analysis was performed. Results: All patients demonstrated lower postoperative symptom scores at six months. Mean reductions were observed for erythema (71.4 percent), burning sensation (61.1 percent) and pruritus (57.1 percent). Local discomfort decreased by 33.3 percent. Mean scar pliability scores increased from 2.2 to 3.2. No postoperative complications, such as infection, fat necrosis or oil cyst formation, were recorded. All patients completed the six month follow up. Conclusions: In this small pilot observational study, autologous fat grafting was well tolerated and associated with descriptive improvement of patient-reported symptoms and scar pliability in irradiated postmastectomy chest wall tissue. These findings suggest a potential symptomatic benefit of fat grafting, while larger studies with objective imaging and histological correlation are required to confirm efficacy and durability.

## 1. Introduction

Radiotherapy is a cornerstone in breast cancer management, effectively reducing local recurrence and has been associated with improvements in some studies [1]. However, its long-term impact on soft tissues frequently leads to complications such as dermal fibrosis, loss of elasticity, pigmentation changes, chronic pain, and delayed wound healing [2]. These radiation-induced sequelae provide considerable obstacles in reconstructive surgery, especially when attempting to restore pliability, volume, and form to previously irradiated chest wall tissues [3].

Autologous fat grafting, often known as lipofilling, is gaining popularity because of its ability to restore volume and regenerate tissue simultaneously [4]. Beyond its original purpose for volume augmentation, fat grafting has shown advantages in improving skin quality, decreasing fibrosis, and boosting vascularity in irradiated tissues [5,6,7]. Previous research indicates that autologous fat grafting may promote angiogenesis, modulate fibrotic processes, and facilitate remodeling of the extracellular matrix. These mechanisms provide a biological rationale for the observed clinical effects, although the present study did not incorporate targeted stem-cell enrichment. In contrast, cell-assisted lipotransfer (CAL), which supplements fat grafts with adipose-derived stem cells (ADSCs), has been shown to enhance graft survival and improve aesthetic outcomes [8,9,10,11,12].

Despite encouraging evidence, clinical evaluations of lipofilling in irradiated breast reconstruction are not standardized. Most studies focus on imaging or volumetric goals, but patient-centered complaints such as pruritus, local discomfort, erythema, and scar stiffness are seldom assessed [13,14]. Structured, symptom-based clinical examinations are still infrequent.

Patient-reported symptoms such as pruritus, burning sensation, erythema, and scar stiffness directly reflect the functional and aesthetic outcomes of breast reconstruction. These subjective manifestations correlate with local tissue fibrosis, perfusion, and elasticity, making them practical and clinically relevant indicators of regenerative success following lipofilling.

With an emphasis on scar modification and symptom relief, the current study attempts to assess the clinical effectiveness and safety of autologous fat grafting in irradiated postmastectomy locations. By applying a structured scoring system, this pilot observational study seeks to contribute evidence on the practical, symptomatic benefits of lipofilling as a regenerative strategy in complex reconstructive scenarios.

In contrast to cell-assisted lipotransfer (CAL), the technique used in this study consisted solely of standard autologous fat grafting without isolation, concentration or reinjection of adipose-derived stem cells. To the best of our knowledge, this is the first pilot observational study focusing on patient-reported symptom improvement following autologous fat grafting in irradiated postmastectomy sites.

The aim of this study was to evaluate the clinical efficacy and safety of autologous fat grafting in improving radiation-induced symptoms and scar quality in postmastectomy patients.

## 2. Materials and Methods

### 2.1. Patient Selection

Five female patients over the age of 18 were included. All had a history of radical mastectomy followed by adjuvant radiotherapy for breast cancer.

Inclusion criteria: A minimum of 6 months since the last radiotherapy session; no evidence of active malignancy or recurrence; presence of radiation-induced symptoms such as pruritus, pain, or scar retraction; and an indication for autologous fat grafting as part of their reconstructive protocol.

Exclusion criteria: uncontrolled systemic diseases, active infections, and previous lipofilling in the same area.

This was a single-center pilot observational study designed to generate preliminary clinical data and to guide future controlled trials.

Study period and design: This pilot observational study was conducted between January and October 2023 at the Plastic Surgery Department, “Pius Brînzeu” Emergency County Hospital, Timișoara, Romania. All surgical procedures and evaluations were performed by the same surgical team to ensure procedural consistency and reduce inter-operator variability.

Anesthesia: All fat grafting procedures were performed under general anesthesia with standard intraoperative monitoring.

### 2.2. Clinical Evaluation

Clinical follow-up was conducted by two physicians, each with a minimum of ten years of experience in reconstructive and plastic surgery. Evaluations were performed at three postoperative time points: 2 weeks, 1 month and 6 months.

### 2.3. Evaluation Scores

Two scoring systems were employed to assess the patients:

- Objective score—*Clinicians* were responsible for assessment of the corresponding scoring metrics, with the final score calculated as the mean of the values recorded by the two evaluators. This methodology was deliberately chosen to ensure the most accurate and reliable assessment of the measured parameters.
- Subjective score—Determined based on *patients’* self-reported assessments. Descriptive symptom scoring using the standardized 0–5 scale was carried out only at baseline and at the 6-month visit. The interim evaluations (2-week and 1-month visits) were conducted to assess wound healing, fat graft stability and early postoperative complications, but no symptom scores were collected at these time-points. Each patient underwent clinical assessment immediately before and at 6 months following the lipofilling procedure. Five clinical domains were evaluated: pruritus, local discomfort, burning sensation, erythema, and subjective scar quality. Symptoms were rated using a standardized 0–5 scale, where higher scores indicated more severe symptoms, except for scar quality, where higher scores reflected improved pliability and appearance. Pruritus, local discomfort, burning sensation and erythema were scored directly by each patient using the same 0 to 5 scale. The values reported in the Results section for these four symptoms represent the patient reported scores without any physician modification or averaging. Scar pliability was rated by the same two physicians using the same 0–5 scale, where higher scores indicated better pliability and appearance [13]. If the two physicians assigned different values, the final scar pliability score used in the analysis represented the arithmetic mean of their evaluations. No imaging, histologic or perfusion-based assessments were performed, and all clinical outcomes were based exclusively on patient-reported symptoms. Symptom scoring was chosen because patient-reported complaints such as pruritus, burning and scar tightness represent clinically relevant outcomes in irradiated chest wall reconstruction. The 0–5 symptom scale used in this study was developed for clinical use within our department and has not undergone formal psychometric validation.

The selected parameters were chosen to reflect the most clinically relevant patient-reported complaints commonly observed following irradiated chest wall reconstruction, including pruritus, burning sensations, scar tightness, and erythema.

Fat harvesting and injection technique: Fat was harvested using the modified Klein super-wet technique with a 20 min infiltration period. The solution consisted of 500 mL normal saline, lidocaine 0.1% and 6 mL sodium bicarbonate 8.4. No vasoconstrictive agents such as epinephrine were added to the tumescent solution to avoid compromising graft perfusion.

Donor areas included the lower abdomen, flanks, or inner thighs. A 4 mm Mercedes-type cannula and moderate negative-pressure suction were used. The lipoaspirate was washed five times with 0.9% saline solution and decanted for 2 min to remove oil and blood residues. Only the purified middle layer was used. No isolation, concentration, or targeted transfer of adipose-derived stem cells was performed. Standard autologous fat grafting without isolation, concentration, or targeted transfer of adipose-derived stem cells was used. This approach reflects routine clinical practice and minimizes additional biological manipulation in a pilot observational setting. In addition, conventional fat grafting permits the transfer of larger adipose volumes and is commonly applied when volumetric restoration is a primary clinical objective, which was relevant for patients with postmastectomy soft-tissue volume deficiency in the present study.

Prior to fat injection, mechanical release of fibrotic adhesions was performed using a rigid Toledo cannula to create recipient space within the irradiated chest wall tissue.

Fat was injected using a Coleman-type technique with multilayered, low-pressure, retrograde delivery through 50 mL Luer-Lock syringes to ensure uniform distribution and reduce resorption [5,12]. On average, 130–170 mL of fat was grafted per breast. The fat was placed into the irradiated chest wall and beneath the mastectomy scar in small aliquots using a multilayered approach. The volume injected per patient is detailed individually in Table 1.

Postoperative follow-up: Patients were reviewed at 6 months postoperatively for clinical improvement, graft retention, and complications, including oil cysts, fat necrosis, or infection. Patients were advised to avoid pressure on the grafted areas for at least 3 weeks [3]. Prophylactic subcutaneous enoxaparin (40 mg daily) was administered for 5 days postoperatively. This protocol was based on evidence suggesting that heparin may enhance graft survival by reducing fibrin deposition and stabilizing angiogenic factors such as FGF-2 [15,16]. All five patients completed the 6-month clinical assessment. One patient subsequently died of unrelated medical causes after the final evaluation.

Statistical analysis: Given the small sample size and the ordinal nature of the 0–5 symptom scale, only descriptive statistics were calculated. Mean values, standard deviations, minimum and maximum scores, and relative percentage changes were reported. No inferential statistical tests were applied, and individual patient trajectories were reviewed descriptively to account for inter-patient variability.

Ethical approval and informed consent: The study was approved by the Institutional Ethics Committee of Victor Babes University of Medicine and Pharmacy, Timisoara (Approval no. 54/25 November 2022). All procedures were conducted in accordance with the principles of the Declaration of Helsinki. Written informed consent was obtained from all the patients prior to inclusion and for the use of clinical data.

Use of artificial intelligence: No generative artificial intelligence (GenAI) tools were used in the design, data collection, analysis, or interpretation of this study. Language editing assistance was limited to grammar and formatting only.

## 3. Results

All patients had previously undergone mastectomy followed by radiotherapy and presented with atrophic, fibrotic, and inelastic chest wall tissues, with or without scar retraction. Four patients had prior tissue expander placement, which was later removed due to infection; one of these still had an expander in place during the lipofilling procedure and developed wound dehiscence. The mean interval between radiotherapy and fat grafting was 1.9 years. All patients exhibited radiodermatitis of varying severity and reported symptoms of pruritus and burning sensation.

Demographic and procedural characteristics are summarized in Table 1 All patients underwent a single session of fat grafting. No immediate postoperative complications were observed. One patient presented with capsular contracture at the time of the procedure, and another patient died during follow-up due to unrelated causes. Physicians’ assessments of scar pliability are presented in Table 2, while individual patient-reported scores are provided in Table 3.

**Figure 1:**
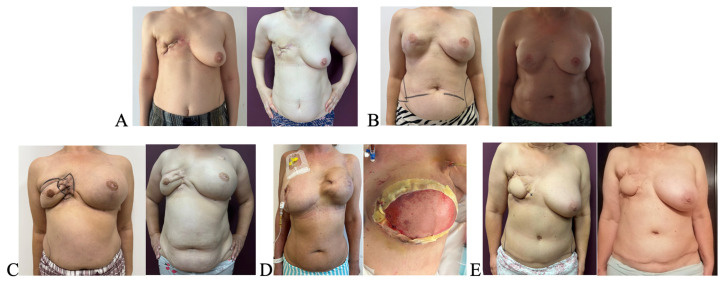
Clinical aspect of irradiated chest walls before autologous fat grafting. (**A**,**C**,**E**) Patients with prior tissue expander removal, treated exclusively with lipofilling. (**B**) Patient with definitive implant in place. (**D**) Patient with previous expander, capsular contracture, and partial dehiscence, treated with lipofilling followed by Deep Inferior epigastric perforator(DIEP) flap reconstruction.

All five patients demonstrated descriptive changes in patient-reported symptoms in the irradiated chest wall following a single session of autologous fat grafting. Visual descriptive changes were noted in skin texture, elasticity, and scar appearance. Improvements were evident both on visual inspection and based on symptom scores. Representative pre- and postoperative clinical aspects are shown in Figure 1.

Quantitatively, the symptom with the greatest improvement was burning sensation, which decreased from a mean of 3.6 to 1.4 (−61%). Pruritus showed a mean reduction from 2.8 to 1.2 (−57%), while erythema decreased from 1.4 to 0.4 (−71%). Local discomfort decreased by 33% (from 3.6 to 2.4), and scar quality improved from a mean of 2.2 to 3.2. Overall, the global mean symptom score across all domains decreased from 2.7 to 1.7 postoperatively (Table 4). Minimum and maximum scores for each symptom domain were also reviewed to illustrate inter-patient variability. Symptom scoring was performed only at baseline and at the 6-month follow-up; interim visits at 2 weeks and 1 month were limited to clinical inspection and postoperative care.

Symptom severity was rated on a 0–5 scale, with higher scores indicating greater symptom burden. All parameters showed lower postoperative symptom scores on the descriptive scale, with the most marked reduction observed in erythema and burning sensation. Scar quality scores increased, indicating higher patient-reported ratings of pliability and appearance on the descriptive scale. One patient died during follow-up due to unrelated medical causes; no events were associated with the procedure.

Subjectively, all patients reported lower symptom burden based on self-reported feedback. Clinicians observed that scars appeared less adherent and that skin texture appeared softer on inspection.

One patient died of unrelated medical causes after completing the 6-month follow-up evaluation; therefore, all symptom scores reported in Table 4 include this patient’s final assessment.

No adverse events, such as fat necrosis, oil cysts, or infections, were observed during follow-up. All patients had an uneventful postoperative course, and no reoperations were needed.

## 4. Discussion

In the present study, reductions in patient-reported symptoms—including burning, pruritus, localized pain, and erythema—were observed. These outcomes, however, must be interpreted with prudence due to the limited cohort size. To address interindividual variability, patient trajectories were examined descriptively.

Prior investigations have delineated multiple biological processes potentially contributing to tissue remodeling following autologous fat grafting, encompassing modulation of fibrotic pathways, remodeling of the extracellular matrix, and the activation of angiogenic signaling cascades, as documented in both experimental and clinical settings [17]. It is important to emphasize that the present study did not incorporate biological, imaging, or histopathological analyses, thereby precluding mechanistic conclusions. The mechanisms referenced herein are intended solely to contextualize the observed descriptive clinical outcomes within this small patient cohort.

Graft quality and delivery precision may be influenced by technical factors in addition to biological and regenerative processes. Experimental ultrastructural studies have shown that larger-diameter cannulas are associated with a higher proportion of intact adipocytes and reduced cellular damage, with potential implications for graft survival [18]. Injection systems also play a role in delivery precision, as syringe size may affect accuracy during fat placement, particularly when large-volume syringes are used, including in the present study [19]. In addition, protocol-driven approaches to autologous fat grafting have been associated with reproducible outcomes in both irradiated and non-irradiated breast reconstruction settings [20].

Clinical studies have reported variable degrees of symptomatic and structural changes after fat grafting in irradiated tissues. Improvements in pliability, texture and subjective comfort have been described in multiple clinical series, although methodological inconsistencies limit comparability [21,22,23,24,25]. Recent clinical experiences have further supported the use of fat grafting techniques in irradiated or surgically altered breast tissues. Pozzi et al. reported favorable aesthetic outcomes using intradermal sharp-needle fat grafting in immediate prepectoral reconstruction, highlighting improvements in contour and soft-tissue quality [26]. Additionally, Susini et al. reviewed advances in capsular contracture management and emphasized the potential value of fat grafting in modulating tissue characteristics in complex reconstructive settings [27]. These reports complement previous clinical findings and provide additional contemporary context for the descriptive observations in the present study. The descriptive symptom trends observed in our cohort are directionally consistent with these reports, but differences in sample size, radiation dose, surgical technique and outcome scoring prevent direct comparison.

Burning and itching are common adverse effects after radiation to the chest wall. In this study, both saw decreases of more than 50% in their postoperative scores on the descriptive scale following lipofilling. Similar symptom trends have been noted in previous clinical series evaluating fat grafting in irradiated tissues [21,22,23,24,25,28,29,30].

No instances of fat necrosis, oil cyst formation, or infectious complications were observed within this cohort, underscoring a favorable immediate safety profile of autologous fat grafting in the context of irradiated tissues. Although the study was not designed to rigorously assess oncologic outcomes and the follow-up duration was insufficient for definitive conclusions, it is notable that no locoregional recurrences or other oncologic events occurred throughout the observation period. While these preliminary findings cannot substitute for long-term surveillance, they provide an initial signal that the procedure does not appear to precipitate early oncologic complications in this highly selected cohort.

The decision to prioritize patient-reported symptom assessments was deliberate, reflecting an awareness that clinically significant complaints—such as pruritus, burning sensations, pain, and scar-related tightness—often remain undetected in imaging-based evaluations. These subjective outcomes, however, are of substantial consequence to patients’ quality of life and functional well-being. By systematically capturing these parameters, the study offers a nuanced perspective on the perceptual and functional impact of autologous fat grafting, which extends beyond structural or radiologic metrics. This approach enables the detection of subtle yet clinically meaningful improvements that might otherwise be overlooked in conventional, imaging-centered assessments.

From a methodological standpoint, the limited sample size precluded formal inferential statistical analyses, including hypothesis testing. Accordingly, descriptive summary statistics were employed, consistent with the conventions of early-phase, exploratory clinical research aimed primarily at hypothesis generation. This framework allowed for the careful examination of individual symptom trajectories, revealing consistent patterns of improvement across the cohort, despite the inherent heterogeneity in prior radiation exposure, elapsed time since irradiation, and baseline degree of fibrosis. Such variability underscores the challenges of interpreting symptom-based outcomes in small patient populations while simultaneously emphasizing the value of detailed, individualized assessments in early-phase clinical investigations.

In the current literature, two notable studies by Sarfati et al. have reported findings comparable to those observed in the present investigation, albeit in larger patient cohorts. These studies provide compelling evidence that preliminary fat grafting to the irradiated chest wall prior to implant reconstruction can enhance skin quality, improve implant coverage, and reduce complication rates. Both studies demonstrated favorable cosmetic outcomes and minimal postoperative complications, with no reports of local cancer recurrence. Collectively, these results align with the observations of the present study, supporting the concept that autologous fat grafting may serve as a valuable preparatory intervention in irradiated tissues, contributing to improved functional and aesthetic outcomes while mitigating surgical risk [31,32].

In summary, while the present study is constrained by cohort size, follow-up duration, and the absence of objective tissue-level assessments, it provides a high-resolution depiction of patient-centered outcomes following autologous fat grafting. The observed symptomatic improvements, systematically documented and analyzed descriptively, offer meaningful insights into the potential functional and perceptual benefits of this intervention, serving as a foundation for hypothesis-driven research and advancing the understanding of patient-centered endpoints in reconstructive care.

## 5. Limitations and Future Perspectives

This investigation is inherently constrained by several methodological limitations that must be acknowledged. Foremost, the absence of a control group precludes any formal comparative analysis, while the exceedingly small cohort (*n* = 5) severely restricts the external validity of the findings. Furthermore, the study relied exclusively on patient-reported symptom assessments, without corroboration from imaging or histopathological evaluations, limiting the capacity to draw mechanistic or objective conclusions regarding tissue alterations. The brevity of the follow-up period further compounds these limitations, as no advanced diagnostic modalities—such as high-resolution ultrasonography, volumetric MRI, or elastography—were employed to quantify structural or functional changes. Patient heterogeneity introduces additional complexity, as variations in prior radiation dosage, time elapsed since irradiation, and degree of fibrosis may have influenced individual symptom trajectories. While prior investigations have employed enriched methodological approaches—including histological analyses and adipose-derived stem cell augmentation—to explore the effects of autologous fat grafting [10,11], such strategies were beyond the scope of the present study, which focused exclusively on clinical and patient-reported outcomes.

Notwithstanding these constraints, the application of a systematic symptom scoring approach and the observation of consistent trends in improvement support the value of this study as an exploratory endeavor, generating hypotheses for subsequent research.

Future investigations should prioritize larger, prospective cohorts with appropriate control arms and standardized clinical assessment tools. To rigorously characterize tissue remodeling following fat grafting in irradiated tissues, incorporation of objective modalities—such as high-resolution ultrasound, elastography, MRI volumetrics, and histological correlation—is essential. Extended follow-up will be critical to ascertain the durability, safety, and potential patterns of graft retention over time. Consequently, the symptom improvements observed herein should be interpreted as descriptive phenomena rather than definitive evidence of therapeutic efficacy.

## 6. Conclusions

In this pilot observational study, standard autologous fat grafting was associated with descriptive improvement in patient-reported symptoms and scar pliability in irradiated postmastectomy chest wall tissue. The procedure was well tolerated, with no observed complications during follow-up. These findings suggest a potential symptomatic benefit of fat grafting in selected patients. Larger studies incorporating objective imaging or histological assessment are required to confirm efficacy and durability.

## Figures and Tables

**Table 1:** Patient demographic and procedural data.

Patient No.	Age (Years)	Mastectomy Type	Time Since Radiotherapy (Months)	Fat Volume Injected (mL)	Adverse Events	Relevant Notes
1	40	Radical	6	120	None	-
2	56	Radical	45	150	Deceased (unrelated)	-
3	52	Radical	17	150	None	-
4	47	Radical	15	150	None	Tissue expander in place, capsular contracture, Underwent DIEP flap
5	59	Radical	18	250	None	-

Note: Patient 2 deceased due to unrelated causes. Patient 4 had a tissue expander in place with capsular contracture.

**Table 2:** Physicians’ assessments of scar pliability at baseline and at six months for all patients.

Patient	Time Point	Scar Pliability—*Physician 1*	Scar Pliability—*Physician 2*	Scar Pliability Final Score (Mean)
**1**	Baseline	4	2	**3**
	6 months	5	3	**4**
**2**	Baseline	2	2	**2**
	6 months	3	3	**3**
**3**	Baseline	3	3	**3**
	6 months	4	4	**4**
**4**	Baseline	3	1	**2**
	6 months	4	2	**3**
**5**	Baseline	1	1	**1**
	6 months	3	1	**2**

**Table 3:** Patient-Reported Subjective Symptom Scores.

Patient	Time Point	Pruritus	Local Discomfort	Burning Sensation	Erythema
**1**	Baseline	4	5	4	2
	6 months	3	3	3	1
**2**	Baseline	3	4	2	1
	6 months	2	3	1	0
**3**	Baseline	2	4	5	2
	6 months	1	3	2	1
**4**	Baseline	1	3	3	1
	6 months	0	2	0	0
**5**	Baseline	4	2	4	1
	6 months	0	1	1	0

**Table 4:** Summary of symptom scores before and after lipofilling (mean ± SD) and percentage change. Note: Higher scores indicate worse symptoms for pruritus, burning sensation, pain and erythema, whereas higher scores indicate better pliability for scar pliability. Accordingly, improvements are expressed as negative percentage changes for symptoms and as a positive percentage change for scar pliability.

Symptom	Before (Mean ± SD)	After (Mean ± SD)	% Change
Pruritus	2.8 ± 1.30	1.2 ± 1.17	−57.1%
Local Discomfort	3.6 ± 1.14	2.4 ± 0.89	−33.3%
Burn Sensation	3.6 ± 1.14	1.4 ± 1.14	−61.1%
Erythema	1.4 ± 0.55	0.4 ± 0.55	−71.4%
Scar Quality	2.2 ± 0.84	3.2 ± 0.84	+45.5%

## Data Availability

The data presented in this study are available on request from the corresponding author. The data are not publicly available due to privacy and ethical restrictions.

## Abbreviations

The following abbreviations are used in this manuscript:

ADSCs	Adipose derived stem cells
CAL	Cell assisted lipotransfer
DIEP	Deep inferior epigastric perforator
MRI	Magnetic resonance imaging
PRP	Platelet rich plasma
